# Effect of bronchofiberscopic lavage with budesonide suspension on refractory mycoplasma pneumoniae pneumonia

**DOI:** 10.12669/pjms.38.4.4835

**Published:** 2022

**Authors:** Ya Li, Wei Yang, Xin Wu, Xiaohua Gou

**Affiliations:** 1 Ya Li, Pulmonary and Critical Care Medicine 2^nd^ Department, Baoding First Central Hospital, Baoding071000, Hebei, China; 2 Wei Yang, Pulmonary and Critical Care Medicine 2^nd^ Department, Baoding First Central Hospital, Baoding071000, Hebei, China; 3 Xin Wu, Department of Public Health Section, Baoding First Central Hospital, Baoding071000, Hebei, China; 4 Xiaohua Gou Pulmonary and Critical Care Medicine 2^nd^ Department, Baoding First Central Hospital, Baoding071000, Hebei, China

**Keywords:** budesonide suspension, bronchofiberscopic lavage, refractory pneumonia, mycoplasma pneumonia

## Abstract

**Objectives:**

To investigate the clinical efficacy of budesonide suspension through bronchofiberscopic lavage in the treatment of refractory mycoplasma pneumoniae pneumonia.(RMPP)

**Methods:**

A total of 108 children with refractory mycoplasma pneumoniae pneumonia from June 2018 to October 2020 were randomly divided into two groups: the study group and the control group, with 54 cases in each group. Children in the control group were treated with anti-infective, antitussive, antipyretic routine symptomatic treatment and nebulization of budesonide suspension, while children in the study group were treated with bronchofiberscopic lavage with Budesonide suspension along with standard treatment as in control group. The clinical efficacy, changes in blood gas analysis indexes, levels of inflammatory mediators, time for improvement of clinical symptoms and the incidence of adverse reactions were compared between the two groups.

**Results:**

The efficacy of treatment in the study group was 85.19%, which was higher than 66.67% in the control group (P<0.05). The antipyretic time, antitussive time, lung rales disappearance time and length of hospital stay in the study group were shorter than those in the control group (P<0.05). Moreover, the levels of arterial oxygen partial pressure (PaO_2_) in the study group were higher than those in the control group, while the levels of partial pressure of carbon dioxide (PaCO_2_) and arterial oxygen saturation (SaO_2_) in the study group were lower than those in the control group (P<0.05). The levels of inflammatory mediators interleukin-6 (IL-6), interleukin-4 (IL-4), and interferon-γ (IFN-γ) in the study group were lower than those in the control group (P<0.05), and no significant difference was found in the comparison of the incidence of adverse reactions between the two groups (P>0.05).

**Conclusions:**

Budesonide suspension combined with bronchofiberscopic lavage with Budesonide suspension is a safe treatment regimen that can significantly improve the clinical outcome in children with RMPP. With such a combined treatment regimen, the clinical symptoms of children can be ameliorated, the ventilation function can be enhanced, and the level of inflammatory mediators will be reduced.

## INTRODUCTION

Mycoplasma pneumoniae pneumonia, as an interstitial pneumonia caused by mycoplasma pneumoniae infection, is one of the most common respiratory infections in clinical practice, with an increasing incidence year by year.^1^-^3^ Macrolide antibiotics are currently the preferred treatment drugs for refractory mycoplasma pneumoniae pneumonia (RMPP). However, with the increase of mycoplasma resistance to macrolide antibiotics, conventional macrolide antibiotics alone have poor efficacy in the treatment of RMPP.^4^,^5^ Budesonide, a non-halogenated glucocorticoid, can reduce vascular permeability, inhibit secretion of mucus, and relieve edema and spasm. Budesonide which is inhaled after nebulization can spread to the whole lung and is highly effective to local inflammation.^6^ In recent years, with the maturity of bronchoscopy technology, the therapeutic value of alveolar lavage on infected sites has been widely recognized.^7^ Budesonide suspension is touted to have a repair effect on inflammatory airway injury tissues, with little systemic effect and high safety.^8^ Local administration of budesonide after bronchoscopic alveolar lavage may achieve better efficacy. In this study, the clinical efficacy of bronchofiberscopic lavage combined with budesonide suspension in the treatment of RMPP was investigated, and adverse reactions were observed.

## METHODS

A total of 108 children with RMPP from June 2018 to October 2020 were randomly divided into two groups: the study group and the control group, with 54 cases in each group. Among all the children, there were 32 males and 22 females in the study group, aged 1-12 years, with an average of 7.84±2.16 years. Their body temperature ranged from 37.5-39.8°C, with an average temperature of 38.44±0.73°C, and the course of disease ranged from 7-14d, with an average course of 9.69±1.24d. In the control group, there were 30 males and 24 females, aged 1-14 years, with an average of 7.95±2.33 years. Their body temperature ranged from 37.8-39.7°C, with an average temperature of 38.37±0.82°C, and the course of disease ranged from 8-15d, with an average course of 9.88±1.36. No statistically significant difference was observed in the general information of the two groups (P>0.05).

RMPP was defined as: (1) sputum culture, serum etiology and lung CT suggested mycoplasma pneumoniae pneumonia in children; (2) a sustained fever for 7 days or more and; (3) increasingly severe cough and infiltrates on chest radiographs despite the administration of appropriate macrolide antibiotics.

### Ethical Approval:

The study was approved by the Institutional Ethics Committee of Baoding First Central Hospital at March 10, 2021(No.2021.3.10), and written informed consent was obtained from all participants

### Inclusion criteria:

- Children who meet the diagnostic criteria for RMPP by imaging examination and laboratory-related instrumental examinations;^9^
- Children aged 1-14 years;
- Children whose family members signed the informed consent and approved by the ethics committee of the hospital.

### Exclusion criteria:

- Children who are allergic to the drugs used in this study;
- Children with hereditary blood diseases and mental diseases;
- Children with pulmonary neoplasms or non-infectious interstitial pulmonary diseases;
- Children with a past history of bronchial asthma, immunodeficiency disease, recurrent respiratory infections, and tuberculosis infectious diseases;
- Children with fungal infection;
- Children with severe malnutrition and debilitating physical conditions.

Children in the control group were given conventional treatment, including symptomatic treatment measures such as expectoration, oxygen inhalation, respiratory support, nutritional support, and postural drainage, and were treated with the following drugs for fourteen days according to the sputum culture drug sensitivity test and serum pathogen detection, such as macrolide antibiotic intravenous drip anti-infection, 10mg/(kg·d) azithromycin (Northeast Pharmaceutical Group Shenyang No.1 Pharmaceutical Co., Ltd., State Drug Approval No.: H20000426), 80-160 mg/(kg·d) cephalosporin antibiotic - cefotiam hydrochloride (Hainan Quanxing Pharmaceutical Co.,Ltd., State Drug Approval No.: H20113468), or 30-100 mg/(kg·d) cefuroxime sodium (Guangdong Jincheng Jinsu Pharmaceutical Co., Ltd., State Drug Approval No.: H20065670) or intravenous infusion of 50-100 mg/(kg·d) cephalosporin sodium (Hainan Huluwa Pharmaceutical Group Co., Ltd., National Drug Approval: H20064990), and nebulized budesonide suspension (AstraZeneca Pharmaceutical Co., Ltd., State Drug Approval No.: H20140475). All drugs given without sputum examination or any other serological test? Quinolones can be an alternative medication; Mycoplasma is generally resistant to beta Lactam.

For children in the study group, on the basis of the treatment regimen of the control group. The children were fasted for four to six hour before surgery, and were given lidocaine spray anesthesia in nasal cavity and pharynx 30 minutes before surgery, and the total amount of medication should be controlled within 5-7mg/kg. During the operation, the child was supine, one side of the nasal cavity was inhaled with high concentration of oxygen through the nasal catheter. And the front end of the fibrous bronchus was lubricated and slowly entered the other side of the nasal cavity, then bronchoalveolar lavage was performed. The sputum of the affected lung segment and leaf was sucked up. The saline at 37°C was infused with a perfusion volume of 5-10ml for each segment of the bronchus. After the infusion, suction was performed with negative pressure, and the pulmonary bronchus in the infected area was flushed 3-4 times each time, with the total amount of perfusion normal saline not more than 5ml/kg. Then budesonide suspension was injected into the lesion site. The treatment time of the study group was the same as that of the control group.

### Observation Indexes

### Criteria for curative effect:^10^

After 10d of treatment, the therapeutic effect was evaluated, and the clinical efficacy was divided into four grades: cured, markedly effective, effective and ineffective. Cured: The children’s body temperature returned to normal, lung rales and symptoms disappeared completely, and the lung lesions were 95% absorbed by X-ray examination.

### Markedly effective:

The temperature of the children was normal, the pulmonary symptoms and lung rales basically disappeared, and the lung lesions were 90% absorbed by X-ray examination. Effective: The temperature of the children dropped by more than 1°C, symptoms were alleviated, lung rales were significantly reduced, lung lesions were 80% absorbed by X-ray examination, and inflammation absorption and lesion abnormality were effectively improved.

### Ineffective:

The children’s high fever did not retreat, the symptoms worsened, the X-ray review changed by 20%, or even worsened. (2) Blood gas indexes were analyzed before and after treatment, and arterial partial pressure of oxygen (PaO_2_), partial pressure of carbon dioxide (PaCO2) and arterial oxygen saturation (SaO_2_) were measured. (3) Before and after treatment, 3ml of fasting peripheral venous blood was collected, and anticoagulant blood was collected in an EDTA vacuum. Centrifugation was carried out in a high-speed refrigerated centrifuge at room temperature at 3000r/min for 15 minutes. After serum separation, the levels of interleukin-6 (IL-6), interleukin-4 (IL-4) and interferon γ (IFN-γ) were detected by double antibody sandwich enzyme-linked immunosorbent assay. (4) The improvement of clinical symptoms and signs in the two groups were counted, including the antipyretic time, antitussive time, lung rales disappearance time and total length of hospital stay. (5) The incidence of adverse reactions in the two groups during treatment was observed.

### Statistical Analysis

SPSS23.0 was used for data statistics and analysis. Measurement data were expressed by mean and standard deviation and analyzed by t test; Count data were expressed by frequency and rate and analyzed by chi-square test. P<0.05 indicates a statistically significant difference.

## RESULTS

The effective rate of treatment for children in the study group was 85.19%, which was higher than 66.67% in the control group (P<0.05). Table-I.The level of PaO_2_ in the two groups was higher than that before treatment, and that in the study group was higher than that in the control group, with a statistically significant difference (P<0.05). The levels of PaCO_2_ and SaO_2_ in the two groups were lower than before treatment, and that in the study group was higher than that in the control group, with a statistically significant difference (P<0.05). Table-II.

**Table-I:** Comparison of clinical efficacy of children [n (%)].

Group	n	Cured	Markedly effective	Effective	Ineffective	Effective rate (n, %)
Study group	54	21	17	8	8	46 (85.19)
Control group	54	13	11	12	18	36 (66.67)
χ^2^						5.066
P						0.024

**Table-II:** Comparison of blood gas index levels between the two groups (X¯±s).

	PaO_2_ (mmHg)		PaCO_2_ (mmHg)		SaO_2_ (%)	

Group	Before treatment	After treatment	Before treatment	After treatment	Before treatment	After treatment
Study group (n=54)	48.33±3.69	81.35±7.93	63.25±6.36	43.54±5.93	53.73±6.78	41.44±5.21
Control group (n=54)	48.69±4.25	67.36±6.91	64.07±5.88	52.08±6.22	53.95±6.24	48.83±4.36
t	0.470	9.774	0.696	7.302	0.175	7.994
P	0.639	<0.001	0.488	<0.001	0.861	<0.001

The levels of IL-6, IL-4, and IFN-γ in the two groups after treatment were lower than those before treatment, and that in the study group was lower than that in the control group, with a statistically significant difference (P<0.05). Table-III. The antipyretic time, antitussive time, lung rales disappearance time and length of hospital stay in the study group were all shorter than those in the control group, with statistically significant differences (P<0.05). Table-IV.No statistically significant difference was observed in the incidence of adverse reactions between the two groups (P>0.05). Table-V.

**Table-III:** Changes in the levels of IL-6, IL-4 and IFN-γ between the two groups (X¯±s).

	IL-6 (pg/ml)		IL-4 (pg/ml)		IFN-γ (pg/ml)	

Group	Before treatment	After treatment	Before treatment	After treatment	Before treatment	After treatment
Study group (n=54)	39.57±5.73	12.35±2.03	102.96±18.54	55.21±10.02	192.98±17.25	110.21±15.32
Control group (n=54)	40.06±5.86	19.78±1.96	103.87±19.25	79.86±11.05	193.65±13.84	149.76±11.02
t	0.439	19.349	0.250	12.144	0.223	15.400
P	0.661	<0.001	0.803	<0.001	0.824	<0.001

**Table-IV:** Improvement time of clinical symptoms and signs in the two groups (X¯±s, d).

Group	Antipyretic time	Antitussive time	Lung rales disappearance time	Length of hospital stay
study group (n=54)	2.59±0.58	5.63±2.07	4.75±1.52	7.98±2.26
Control group (n=54)	3.47±0.68	7.54±2.38	6.25±1.57	9.83±3.45
t	7.235	4.450	5.044	3.296
P	<0.001	<0.001	<0.001	0.001

**Table-V:** Occurrence of adverse reactions in the two groups during treatment.

Group	Sinus Tachycardia	Decreased blood Oxygen Saturation	Vomiting	Gastrointestinal Reaction	Total Incidence n(%)
Study group (n=54)	2	1	1	1	5 (9.26)
Control group (n=54)	1	0	2	1	4 (7.41)
X^2^					0.121
P					0.728

## DISCUSSION

In recent years, the number of refractory or severe cases of mycoplasma pneumoniae pneumonia has increased gradually, with the clinical characteristics of rapid onset, fast progress and relatively migratory courses. Its clinical symptoms range from persistent high fever and cough to reduced breathing sounds with or without rales, and its pulmonary imaging is manifested as large-scale high-density consolidation.^11^-^13^

Antibiotic anti-infective therapy is the preferred routine treatment for mycoplasma infection. However, strain resistance has been enhanced owing to the massive use of antibiotics at the present stage, resulting in the failure of conventional anti-infective treatments to achieve the desired effect, and the slow recovery of clinical symptoms in children.^14^-^16^ Budesonide suspension is a glucocorticoid with potent topical anti-inflammatory effects that can enhance the stability of the lysosomal membrane of endothelial cells and smooth muscle cells, inhibit immune response and reduce antibody synthesis. In this way, the release of allergic active mediators such as histamine can be reduced, the activity can be inhibited, the enzymatic process triggered by the binding of antigen and antibody can be reduced, and the synthesis and release of broncho-constrictive substances can be inhibited, thereby reducing the contraction response of smooth muscles, diminishing the leakage of capillaries, exerting a strong local anti-inflammatory effect, inhibiting the hyperresponsiveness of the airway, reducing the secretion of glands, relieving symptoms, and repairing damaged airways.^17^-^19^ Bronchofiberscopy and alveolar lavage are non-invasive methods developed in recent years. With a soft texture, small lumen and strong light conduction ability, the bronchofiberscope can be easily penetrated into the airway of children, so as to facilitate the observation of the trachea or the opening of the left and right lobes of bronchus and the mucosa. Single or multiple alveolar lavage by bronchoalveolar lavage (BAL) can effectively remove inflammatory secretions of the respiratory tract in children. In combination with antibiotic drugs, mucosal edema in children can be significantly reduced, sputum dissolution can be promoted, and topical anti-inflammatory effect can be intensified, so as to ameliorate clinical symptoms of children.^20^

In this study, budesonide suspension combined with bronchofiberscopic lavage significantly improved the clinical efficacy of RMPP. Studies have shown that the use of intratracheal corticosteroids can not only recruit the lungs, but also alleviate pulmonary inflammation in severe respiratory distress syndrome, which provides support for our study.^21^ Marraro et al. found that children with bronchoalveolar lavage could improve oxygenation index and Pao2/Fio2 more quickly than those with bronchial atomization inhalation under the same medication.^22^ In this study, the levels of PaO2 in the study group were higher than those in the control group, and the levels of PaCO2 and SaO2 were lower than those in the control group, which was consistent with the results of the study. This is mainly related to the fact that bronchofiberscopic lavage can basically remove inflammatory secretions in the trachea of children and relieve airway obstruction. The children treated with budesonide suspension combined with bronchofiberscopic lavage had significantly shorter antipyretic time, antitussive time, lung rales disappearance time and length of hospital stay than those treated with conventional treatment, and their conditions were effectively controlled and clinical symptoms were significantly improved, which was supported by the study results of Tan et al.^23^ There was no significant difference in the incidence of adverse reactions between the two groups during treatment, indicating the high safety of budesonide suspension combined with bronchofiberscopic lavage.

It has been shown in studies^24^ that the expressions of inflammatory factors such as IL-6, IL-4 and IFN-γ in serum of children with RMPP were up-regulated, lymphocytes in infected sites were reduced, neutrophils were increased, and local reactions were aggravated. IL-4 can activate the synthesis of immune proteins, leading to wheezing attacks. IL-6 is a multifunctional cytokine, which has a close bearing on the immune response and the regulation and defense function of the hematopoietic system. It is positively correlated with the severity of infection and inflammation, and is involved in the process of pneumonia infection. IFN-γ is mainly produced by Th1 cells and is involved in regulating the immune function of the body after infection, and can mediate the production of other inflammatory mediators.^25^ Budesonide suspension combined with bronchofiberscopic lavage has been touted for various benefits, such as blocking the release of inflammatory mediators and cytokines, significantly reducing serum IL-6, IL-4, and IFN-γ levels in children with RMPP, diminishing airway inflammation caused by abnormal immune responses, and improving respiratory symptoms in children.

### Limitations of the study:

There are still some shortcomings in this study. First of all, the number of subjects included in this study is limited, so the conclusions drawn may not be very convincing. In addition, we should have set up a control group of conventional treatment combined with bronchofiberscopic lavage with normal saline alone to make the findings more definitive and convincing.

## CONCLUSION

Budesonide suspension combined with bronchofiberscopic lavage is a safe treatment regimen that can significantly improve the clinical efficacy of children with RMPP. With such a combined treatment regimen, the clinical symptoms of children can be ameliorated, the ventilation function can be enhanced, and the level of inflammatory mediators will be reduced.

### Authors’ Contributions:

**YL & XG:** Designed this study and prepared this manuscript, and are responsible and accountable for the accuracy or integrity of the work. **WY:** Collected and analyzed clinical data. **XW:** Significantly revised this manuscript.
